# Anticonvulsant Effect of *Antiaris toxicaria* (Pers.) Lesch. (Moraceae) Aqueous Extract in Rodents

**DOI:** 10.1155/2013/519208

**Published:** 2013-09-18

**Authors:** Priscilla Kolibea Mante, Donatus Wewura Adongo, Eric Woode, Kennedy Kwami Edem Kukuia, Elvis Ofori Ameyaw

**Affiliations:** ^1^Department of Pharmacology, Faculty of Pharmacy and Pharmaceutical Sciences, Kwame Nkrumah University of Science and Technology, Kumasi, Ghana; ^2^Department of Pharmacology, University of Ghana Medical School, University of Ghana, Accra, Ghana; ^3^Department of Biomedical and Forensic Sciences, School of Biological Science, University of Cape Coast, Cape Coast, Ghana

## Abstract

*Antiaris toxicaria* (Moraceae) was evaluated for anticonvulsant activity in rodents. Animal models used include maximal electroshock test (MEST); pentylenetetrazole-induced (PTZ) convulsions; picrotoxin-induced (PCT) convulsions; strychnine- (STR-) and 4-aminopyridine-induced convulsions. Increase in latency to seizures as well as reduction in duration and frequency of seizures indicated anticonvulsant activity. The extract was more effective in all models used except the maximal electroshock test and strychnine-induced convulsions. *Antiaris toxicaria* aqueous extract (200, 400, and 800 mg kg^−1^) significantly (*P* < 0.05 − 0.01) shortened the duration of convulsions in PTZ- and PCT-induced seizures. Delay in the onset of convulsions in the two tests was significant (*P* < 0.001). Reduction in the frequency of seizures was also significant (*P* < 0.05 − 0.001) in both tests. *Antiaris* further delayed the onset of seizures in 4-aminopyridine model while producing 75% protection against death in mice. Diazepam (0.1, 0.3, and 1 mg kg^−1^), carbamazepine (3, 10, and 30 mg kg^−1^), and sodium valproate (100–400 mg kg^−1^) were used as reference anticonvulsant drugs for various models. Flumazenil blocked the effect of the extract in the PTZ test significantly suggesting that *Antiaris toxicaria* may be acting by enhancing the effects of the GABAergic system. *Antiaris toxicaria* aqueous extract therefore possesses anticonvulsant activity.

## 1. Introduction

The plant *Antiaris toxicaria* (family Moraceae) is an indigenous plant common in Ghanaian forests. It is known locally as “foto” or “kyenkyen” in Akan and the bark cloth tree in English [1]. Despite considerable advancements in the treatment of neurological disorders, epilepsy remains a significant therapeutic challenge [2]. Currently available antiepileptic drugs (AEDs) have debilitating adverse effects on cognition and behaviour [3]. These adverse effects are commonly and consistently observed with barbiturates, benzodiazepines, and topiramate [4, 5]. This problem is further compounded by polypharmacy which characterizes treatment of epilepsy. These problems are known to prevail more in developing countries due to lack of facilities for proper diagnosis and treatment along with monitoring of AED serum levels [6, 7].

Natural products and plants already used in traditional medicine can be a good place to start in the search for safer and more effective options. Numerous plants used for the treatment of epilepsy traditionally have been shown to be potent in models of epilepsy and several such plants remain to be scientifically validated [8]. *Leonotis leonurus, Delphinium denudatum, Mimosa pudica, *and* Synedrella nodiflora* are but a few examples [8–10]. Preliminary screening of the aqueous extract of *Antiaris toxicaria* revealed significant anticonvulsant effect in pentylenetetrazole-induced seizure test [11]. Hence, this study seeks to further explore the extract's potential as an anticonvulsant.

## 2. Materials and Methods

### 2.1. Plant Material

Stem bark of *Antiaris toxicaria* was harvested from the KNUST campus, Kumasi, and authenticated at the Pharmacognosy Department of the Faculty of Pharmacy, KNUST, Kumasi, Ghana. A voucher specimen (KNUST/HM1/011/S007) was retained in the herbarium.

### 2.2. Preparation of *Antiaris toxicaria* Aqueous Extract

Dried stem bark was milled into powder using a commercial grinder. The coarse powder (431 g) was extracted by cold maceration with distilled water as solvent at room temperature for five days. Filtrate was oven-dried to obtain a yield of 23.40% w/w of *Antiaris toxicaria* aqueous extract (AAE).

### 2.3. Animals

Male ICR mice and Sprague-Dawley rats weighing between 20 and 25 g and 120 and 145 g, respectively, were obtained from Noguchi Memorial Institute for Medical Research, Accra, Ghana, and kept in the departmental animal house. Animals were maintained under laboratory conditions of temperature, humidity, and light, housed in stainless steel cages (34 × 47 × 18 cm^3^), and allowed access to water and food *ad libitum*. The animals were assigned to treatment groups of eight to ten animals randomly. All animals were handled in accordance to the Guide for the Care and Use of Laboratory Animals [12] and experiments were approved by the Faculty Ethics Committee.

### 2.4. Drugs and Chemicals

Diazepam (DZP), pentylenetetrazole (PTZ), picrotoxin (PCT), 4-aminopyridine (4-AP), and strychnine (STR) were purchased from Sigma-Aldrich Inc., St. Louis, MO, USA. Flumazenil was obtained from APP Pharmaceuticals, LLC, Schaumburg, IL, USA.

### 2.5. Pentylenetetrazole-Induced Seizures

The method as described by Vellucci and Webster, 1984, and Moezi et al., 2011, [13, 14] was used. The plant extract was administered at doses of 200, 400, and 800 mg kg^−1^ body weight orally. Intraperitoneal (i.p) injection of diazepam (0.1, 0.3, and 1 mg kg^−1^) was used as reference anticonvulsant drug. Animals were pretreated with the plant extract thirty minutes and diazepam fifteen minutes before administration of pentylenetetrazole (PTZ) 85 mg kg^−1^ subcutaneously. Control animals were pretreated with distilled water (10 mL kg^−1^, *p.o*.). The onset of, total duration as well as frequency of clonic seizures were measured within a thirty minute period.

### 2.6. Picrotoxin-Induced Seizures

Animals received AAE at doses of 200, 400, and 800 mg kg^−1^ (*p.o.*) body weight. Picrotoxin (PCT) was injected intraperitoneally at a dose of 3 mg kg^−1^ fifteen minutes after pretreatment with diazepam and thirty minutes after AAE. Diazepam (0.1, 0.3, and 1 mg kg^−1^) served as positive control. Control animals received distilled water (10 mL kg^−1^, *p.o*.). Anticonvulsant activity was scored similarly to that stated in the PTZ test [13, 15].

### 2.7. Maximal Electroshock Test (MEST)

Tonic convulsions of hind limb extremities of mice were induced using electrical current (50 mA, 60 Hz, and 0.2 seconds) via ear clip electrodes. Control group animals received distilled water orally (10 mL kg^−1^, *p.o*.). Carbamazepine at doses of 3, 10, and 30 mg kg^−1^ orally served as reference anticonvulsant. AAE was tested at doses of 200, 400, and 800 mg kg^−1^ orally. Convulsions were induced thirty minutes after pretreatment. Latency to and total duration of hind limb tonic extension were recorded [16].

### 2.8. Strychnine-Induced Convulsions

This test was carried out as described by Bogdanov et al. in 1997 [17]. AAE was administered orally at doses of 200, 400, and 800 mg kg^−1^ body weight. Strychnine was injected subcutaneously at a dose of 2 mg kg^−1^ thirty minutes after AAE administration. Standard anticonvulsant employed was diazepam (0.1, 0.3, and 1 mg kg^−1^, i.p). Animals were observed via video recording. The onset of, decrease in the total duration plus frequency of tonic convulsions were taken as indication of anticonvulsant activity.

### 2.9. 4-Aminopyridine-Induced Convulsions

Animals received AAE orally at doses of 200, 400, and 800 mg kg^−1^ body weight. 4-Aminopyridine was dissolved in normal saline and injected subcutaneously at a dose of 10 mg kg^−1^ body weight thirty minutes after drug treatments. Control animals were pretreated with normal saline (10 mL kg^−1^) and sodium valproate at 100, 200, and 400 mg kg^−1^ served as positive control. Hind limb tonic extensions and decrease in the total duration of convulsions were recorded. Method as described by Morales-Villagrán and Tapia, 1996, [18] was used with slight modifications. 

### 2.10. Effect of AAE on GABA_A_

In order to investigate the mechanism of action of AAE as an anticonvulsant, mice were treated with flumazenil, a benzodiazepine antagonist (1 mg kg^−1^, i.p) fifteen minutes before the administration of AAE (400 mg kg^−1^, *p.o.*). Thirty (30) minutes later, convulsions were induced with pentylenetetrazole at 85 mg kg^−1^, intraperitoneally. Other groups of animals received AAE or flumazenil or diazepam (0.3 mg kg^−1^, i.p) only. Animals were observed for thirty minutes after treatment via video recording for latency and duration of convulsions. 

### 2.11. Data Analysis

Analysis of variance (ANOVA) followed by Newman-Keuls' *post hoc* test was used to determine significant differences between means. Two-way ANOVA followed by Bonferroni test was used in the flumazenil experiment. In the 4-aminopyridine seizure test, the Kaplan-Meier method was used in estimating survival relative to time and survival differences were analyzed with the log-rank test. Statistical analyses were carried out with Graph Pad Prism Version 5.0 (GraphPad Software, San Diego, CA, USA) and SigmaPlot Version 11.0 (Systat Software, Inc., Chicago, USA). Values were presented as mean ± S.E.M., and *P* < 0.05 was considered significant.

## 3. Results 

### 3.1. Effects in Pentylenetetrazole-Induced Seizures

**Figure 1:**
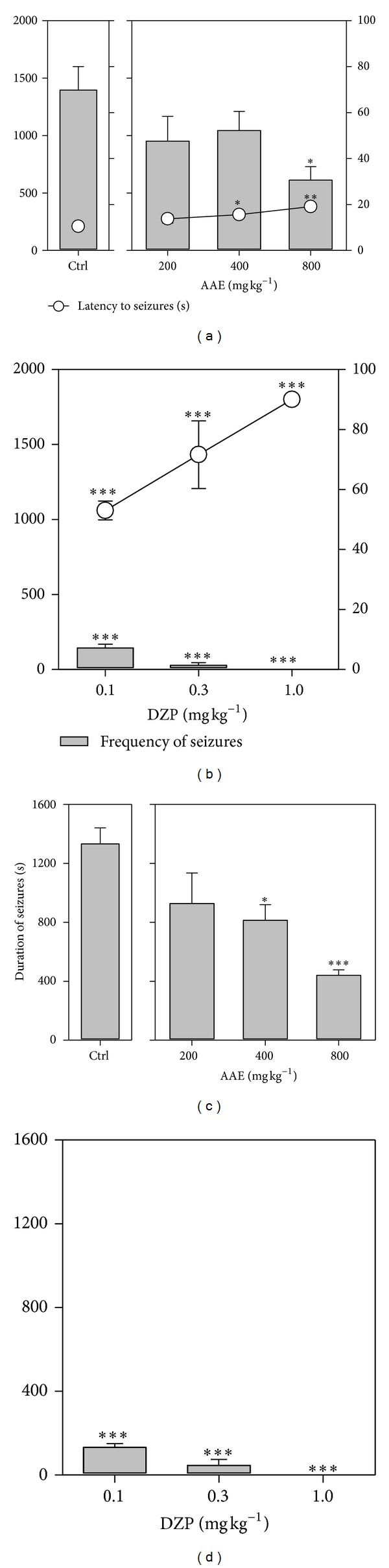
Effects of AAE (200, 400, and 800 mg kg^−1^, *p.o.*) and diazepam (0.1, 0.3, and 1.0 mg kg^−1^, i.p.) on the latency and frequency of seizures ((a) and (b)) and duration of convulsions ((c) and (d)) in PTZ-induced seizures. Data are presented as mean ± S.E.M. (*n* = 8). *P < 0.05, **P < 0.01, and ***P < 0.001 (one-way analysis of variance followed by Newman-Keuls' *post hoc* test).

Pentylenetetrazole (85 mg kg^−1^, s.c.) produced myoclonic jerks in all mice pretreated with the distilled water. AAE (200–800 mg kg^−1^) produced a significant (*P* = 0.0021;  *F*_3,16_ = 7.671; Figure 1(a)) dose-dependent increase in time taken to the onset of clonic seizures. In the extract-treated animals, frequency of seizures was also decreased nondose-dependently, and only the 800 mg kg^−1^ dose was significant (*P* = 0.0511; *F*_3,16_ = 3.213; Figure 1(a)). AAE produced significant (*P* = 0.0018; *F*_3,16_ = 8.005; Figure 1(c)) dose-dependent decrease in the total duration of convulsions in all animals pretreated with the various doses of the extract. The reference anticonvulsant diazepam (0.1–1.0 mg kg^−1^, i.p) also profoundly delayed the onset of myoclonic jerks, decreased frequency of jerks and significantly (*P* < 0.0001; *F*_3,16_ = 125.8; Figures 1(b) and 1(d)) antagonized PTZ-induced seizures.

### 3.2. Effects in Picrotoxin-Induced Seizures

**Figure 2:**
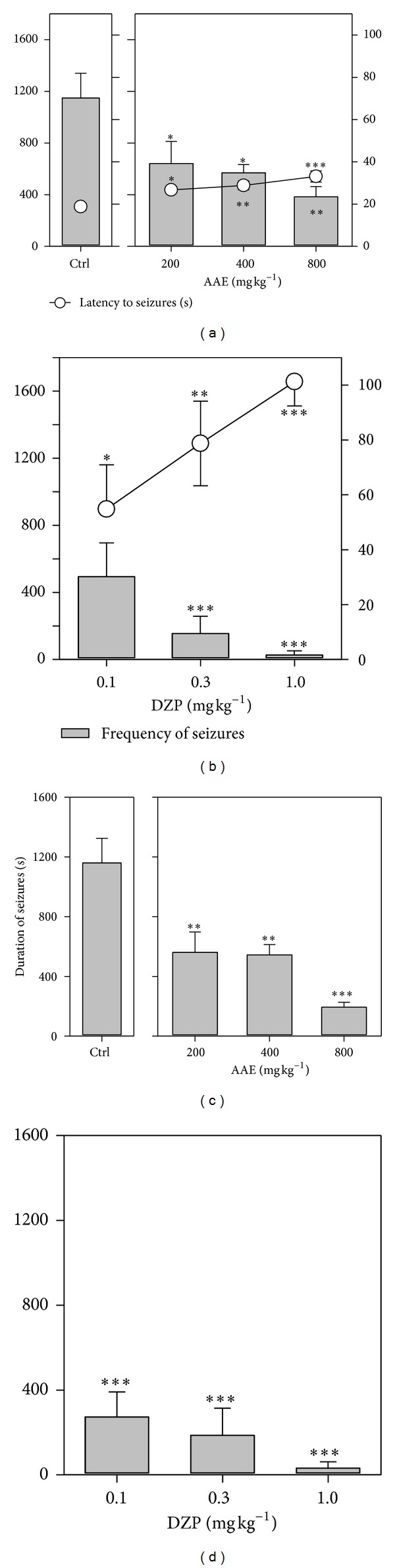
Effects of AAE (200, 400, and 800 mg kg^−1^, *p.o.*) and diazepam (0.1, 0.3, and 1 mg kg^−1^, i.p.) on the latency and frequency of seizures ((a) and (b)) and duration of convulsions ((c) and (d)) in picrotoxin-induced seizures. Data are presented as mean ± S.E.M. (*n* = 8). *P < 0.05,**P < 0.01, and ***P < 0.001compared to vehicle-treated group (one-way analysis of variance followed by Newman-Keuls' *post hoc* test).

Picrotoxin (3 mg kg^−1^, i.p) produced clonic seizures in all mice pretreated with the distilled water. AAE (200–800 mg kg^−1^) produced a dose-dependent increase in latency to seizures which was significant (*P* = 0.0009; *F*_3,16_ = 9.108; Figure 2(a)) at all doses. The frequency of seizures was also significantly (*P* = 0.0081*; F*_3,16_ = 5.591; Figure 2(a)) decreased dose-dependently. AAE produced significant (*P* = 0.0002*; F*_3,16_ = 12.53; Figure 2(c)) dose-dependent decrease in the total duration of seizures in all animals. The reference anticonvulsant diazepam (0.1–1.0 mg kg^−1^, i.p.) also profoundly delayed the onset of myoclonic jerks, decreased frequency of jerks and significantly antagonised (*P* < 0.0001*; F*_3,16_ = 17.72; Figures 2(b) and 2(d)) PCT-induced seizures. 

### 3.3. Effects in Maximal Electroshock Test

**Figure 3:**
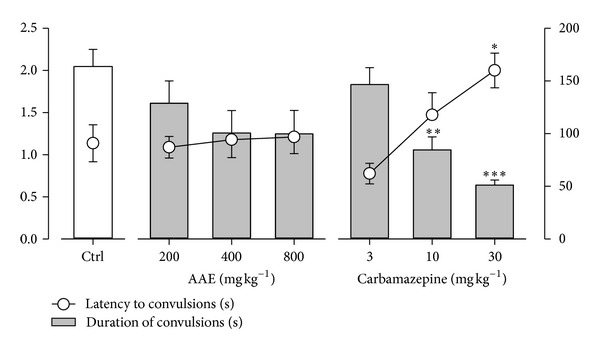
Effects of AAE (200, 400, and 800 mg kg^−1^, *p.o*.) and carbamazepine (3, 10 and 30 mg kg^−1^, *p.o.*) on the latency to and duration of convulsions in the MEST. Each point and column represents mean ± S.E.M. (*n* = 8).  *P < 0.05,  **P < 0.01  ***P < 0.001 compared to vehicle-treated group (one-way analysis of variance followed by Newman-Keuls' *post hoc* test).

Electroshock produced hind limb tonic extensions (HLEs) in all mice. The extract did not affect the latency to onset of hind limb tonic extensions and duration of convulsions significantly (Figure 3). The reference anticonvulsant carbamazepine (3, 10, and 30 mg kg^−1^, *p.o.*), however, delayed the onset of HLEs significantly (*P* = 0.0054; *F*_3,16_ = 6.193; Figure 3) and decreased the total duration of electroshock-induced convulsions. 

### 3.4. Effect in Strychnine-Induced Convulsions

**Figure 4:**
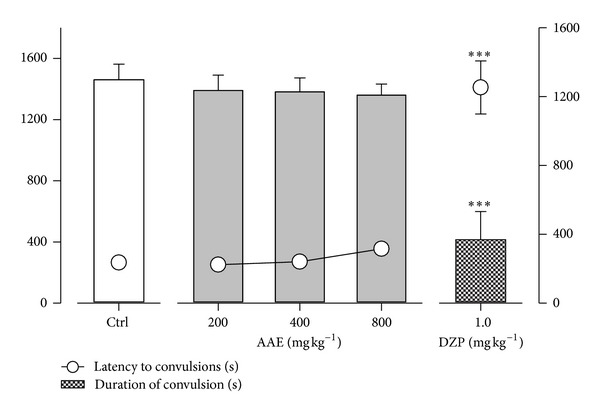
Effects of AAE (200, 400, and 800 mg kg^−1^, *p.o.*) and diazepam (1 mg kg^−1^, i.p.) on the latency to and duration of convulsions in strychnine-induced seizures. Data are presented as mean ± S.E.M. (*n* = 10). ***P < 0.001 compared to vehicle-treated group (one-way analysis of variance followed by Newman-Keuls' *post hoc* test).

Strychnine produced hind limb tonic extensions (HLEs) in all mice. AAE did not affect the latency to onset of hind limb tonic extensions and duration of convulsions significantly (Figure 4). The reference anticonvulsant diazepam (1 mg kg^−1^, i.p), on the other hand, delayed the latency to convulsions significantly (*P* < 0.0001; *F*_4,20_ = 36.60; Figure 4). The duration of convulsions was also significantly (*P* < 0.0001; *F*_4,20_ = 14.31; Figure 4) decreased.

### 3.5. Effects in 4-Aminopyridine-Induced Convulsions

**Figure 5:**
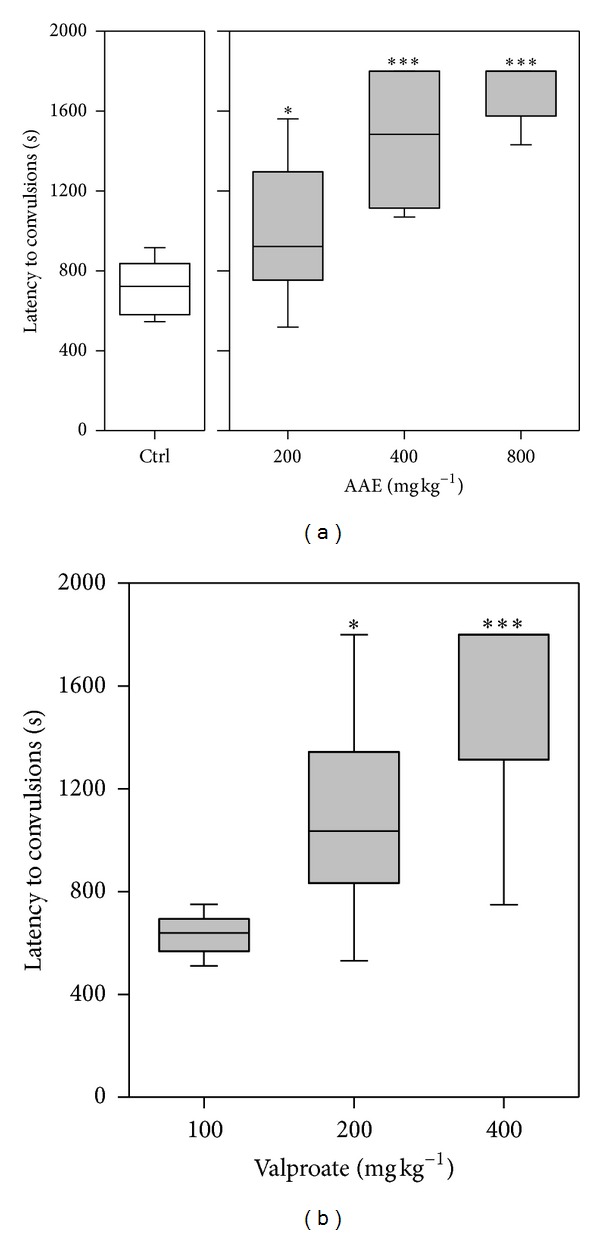
Effects of AAE (200, 400, and 800 mg kg^−1^, *p.o*.) and sodium valproate (100, 200, and 400 mg kg^−1^, *p.o.*) on the latency to convulsions in 4-aminopyridine-induced seizure test. Data are presented as group mean ± S.E.M. (*n* = 8). The lower and upper margins of the boxes represent the 25th and 75th percentiles, with the extended arms representing the 10th and 90th percentiles, respectively. The median is shown as a horizontal line within the box. *P < 0.05,***P < 0.001 compared to vehicle-treated group (one-way analysis of variance followed by Newman-Keuls *post hoc *test).

**Figure 6:**
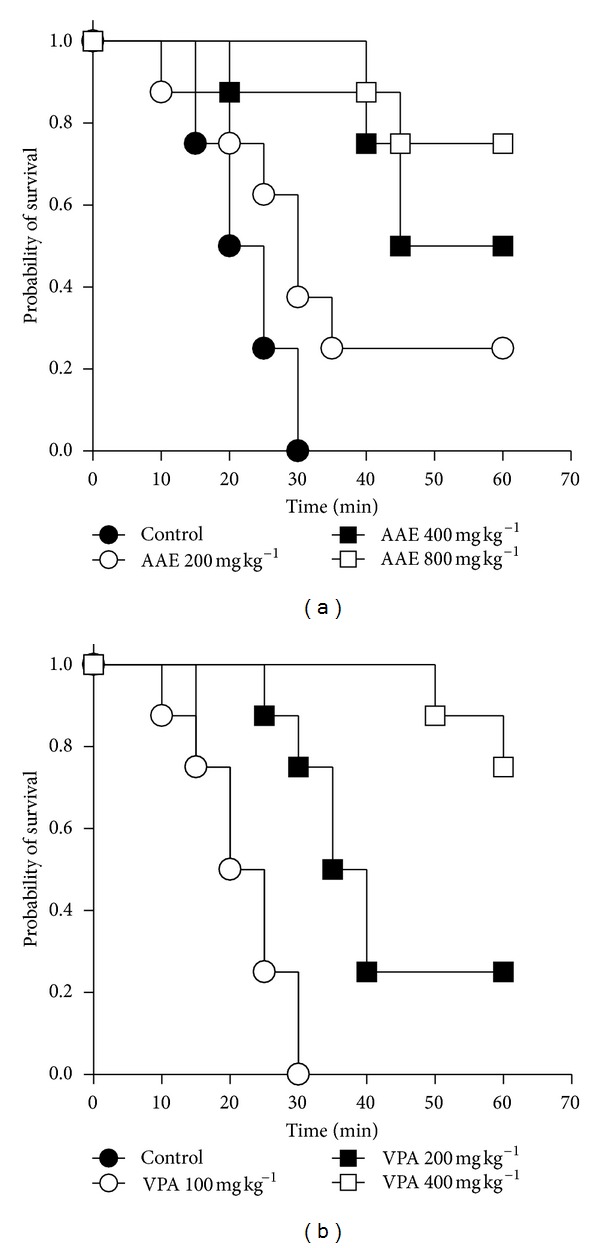
Kaplan-Meier estimates of overall survival of animals treated with (a) AAE (200, 400, and 800 mg kg^−1^) and (b) sodium valproate (100, 200, and 400 mg kg^−1^) in the 4-aminopyridine seizure test over one-hour observation period (*n* = 8).

4-Aminopyridine (10 mg kg^−1^, i.p.) produced hind limb tonic extensions in all animals. The extract produced a significant (*P* < 0.0001;  *F*_3,28_ = 22.02; Figure 5(a)) increase in time taken to the onset of convulsions. Sodium valproate (100–400 mg kg^−1^) also significantly (*P* < 0.0001; *F*_3,28_ = 17.61; Figure 5(b)) delayed the onset of convulsions. The extract significantly (*P* = 0.0004,  *χ* ^2^(df = 3) = 18.11; Figure 6(a)) improved survival of the animals after induction of convulsions. The test for trend presented significant (*P* < 0.0001, *χ* ^2^(df = 1) = 15.79) effect of the treatment groups on median survival indicating a linear trend. Sodium valproate also produced similar effects on survival (*P* < 0.0001, *χ* ^2^(df = 3) = 21.23; Figure 6(b)) and linear trends (*P* < 0.0001,  *χ* ^2^(df = 1) = 19.17). Survival curves show a decrease in probability of survival with time. However, probability of survival increased with increasing dose.

### 3.6. Effects of Flumazenil

**Figure 7:**
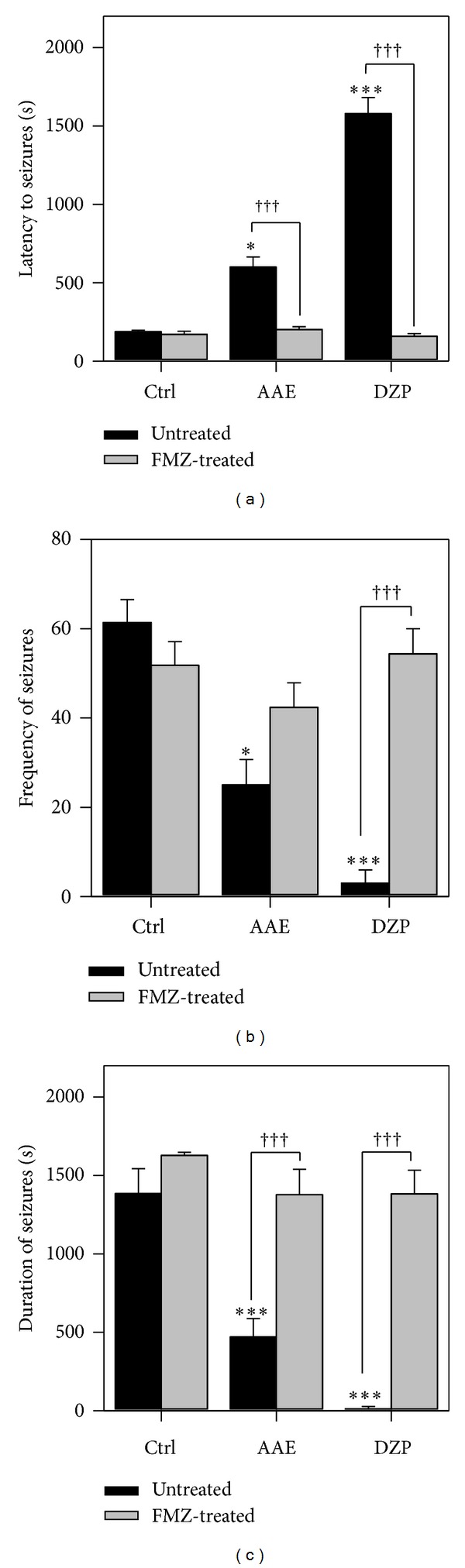
Effect of flumazenil on the latency (a), frequency (b), and duration of seizures (c) of AAE (400 mg kg^−1^, *p.o*.) and diazepam (0.3 mg kg^−1^, i.p.) (c) in PTZ-induced seizures. Data are presented as mean ± S.E.M. (*n* = 8). *P < 0.05,**P < 0.01, and ***P < 0.001 compared to vehicle-treated group (one-way analysis of variance followed by Newman-Keuls' *post hoc* Test). ^†††^ *P* < 0.001 (two-way ANOVA followed by Bonferroni posttest).

Flumazenil significantly (*P* = 0.0009; *F*_1,8_ = 26.09; Figure 7(c)) reversed the reduction in duration of seizures produced by the extract. A significant reversal (*P* = 0.0003; *F*_1,8_ = 37.51; Figure 7(a)) was also obtained for the latency to seizures. The frequency of seizures as compared to the control showed no significant effect. Effects of diazepam were significantly (*P* < 0.0001; Figures 7(a), 7(b), and 7(c)) reversed by flumazenil.

## 4. Discussion 

The outcome of this study provides evidence that the aqueous extract of the stem bark of *Antiaris toxicaria* possesses anticonvulsant activity. The ability of AAE to delay the onset of convulsions and/or shorten the duration of convulsions was considered an indication of anticonvulsant activity. Judging from the data obtained, the plant extract exhibited anticonvulsant activity in the PTZ test just like diazepam which can be due to action on GABA system [19–21]. The exact mechanism by which PTZ produces seizures is not well understood. It, however, has been shown to be due to inhibition and/or attenuation of GABAergic neurotransmission [22, 23]. It is therefore likely that AAE produces its anticonvulsant effect directly or indirectly by enhancing GABAergic neurotransmission in the brain. The PTZ test models human generalized and absence seizures [24, 25]. Hence, the extract may be effective in managing such conditions.

Picrotoxin is a GABA_A_-receptor antagonist [26, 27]. GABAergic ionotropic receptors can mediate both pre- and post-synaptic inhibition. Pre-synaptic inhibition mediated by GABA often leads to inhibition of neurotransmitter release from the excitatory arm [28]. PCT-induced seizures which are due to the decreased GABA_A_-receptor-mediated inhibition in turn promote the excitatory arm of the CNS mainly mediated by glutamate [20, 22]. The extract being effective in the PCT-induced seizure test points to a more specific action on GABA-mediated neurotransmission. This has been further confirmed by the use of flumazenil to reverse the anticonvulsant properties of AAE in the PTZ-induced seizure model. Flumazenil is a benzodiazepine antagonist at the GABA_A_ receptor possessing the ability to reverse anticonvulsant effects and the accompanying alterations in extracellular glutamate concentration [29]. The specificity of flumazenil to this receptor has been proven as several authors have demonstrated that the antagonist has no effect on diazepam's action on voltage-dependent Na^+^ channels [29–31].

4-Aminopyridine is a potassium ion channel antagonist. AAE significantly and dose-dependently increased the latency to convulsions induced with 4-aminopyridine. This may be due to activation of potassium channels or conductance. GABAergic receptor activation can result in enhancement of potassium conductance. AAE may therefore be acting either directly or indirectly to enhance potassium ion conductance. Retigabine is currently the only approved drug for the treatment of epilepsy which functions through activation of potassium currents [32–34] and has promise in the treatment of pharmacoresistant epilepsy. It is also known that 4-aminopyridine stimulates the release of neurotransmitters, including glutamate, in numerous preparations such as brain slices [35–38] and the neuromuscular junction [39]. Furthermore, it has been recently found by a microdialysis procedure *in vivo* that glutamate is by far the amino acid predominantly released by 4-aminopyridine in the striatum [18]. Therefore, due to the well-recognized function of excitatory amino acid receptors in convulsive and excitotoxic mechanisms [40], it seems feasible that the excess glutamate released may be involved in the excitatory effects. This indicates that the extract may be acting, in addition to the above, by modulating the release of glutamate. From the pattern of survival curves, it can be inferred that the dose of the extract does affect survival rate offering significant protection against death from 4-aminopyridine-induced convulsions. This was evident from the log-rank analysis which indicated significant differences between survival curves for each dose level. Median survival time ranged from 30 min and death may be prevented totally at the highest dose of AAE. The exact mechanism by which the extract offers this protection may be linked to its ability to reduce excitotoxic effects mediated by glutamate release. Sodium valproate also increased median survival time and was more effective than the extract.

The extract showed no effect in the MEST at all doses. This indicates that it is not effective in partial and generalized tonic seizures [41] and is unable to prevent seizure spread [8]. It was also ineffective in strychnine-induced convulsions. Strychnine induces convulsions by antagonizing competitively the postsynaptic inhibitory effects of glycine [42]. The fact that AAE produced no protective effects against strychnine-induced convulsions suggests it does not interact with the glycine-mediate inhibitory pathway.

## 5. Conclusion

The results presented here indicate that the aqueous extract of *Antiaris toxicaria* exhibits anticonvulsant activity, possibly through GABA-mediated inhibition and inhibition of glutamate mediated excitation via activation of potassium ion channels.
